# Characterizing Root Morphology, Water and Nitrogen Uptake of Fibrous-Root and Taproot Crops Under Transparent Soil

**DOI:** 10.3390/plants14213369

**Published:** 2025-11-04

**Authors:** Sen Li, Jinjie Fan, Djifa Fidele Kpalari, Kanghu Li, Shoutian Ma

**Affiliations:** 1 Institute of Farmland Irrigation, Chinese Academy of Agriculture Sciences/Key Laboratory of Crop Water Use and Regulation, Ministry of Agriculture and Rural Affairs, Xinxiang 453002, China; lisen18@caas.cn (S.L.); dkpalari@gmail.com (D.F.K.); lkh19980430@163.com (K.L.); 2 School of Water Resource, North China University of Water Resources and Electric Power, Zhengzhou 450046, China

**Keywords:** transparent soil, hydroponics, root morphology, water consumption, nitrogen uptake

## Abstract

Root morphology and uptake capacity are increasingly investigated as indicators of crop performance, yet their characterization remains challenging in laboratory. Soil or sand are opaque to most forms of radiation, while transparent medium fails to provide soil-relevant characteristics. Transparent soil (TS) is specifically designed to support root growth in the presence of air, water, and nutrients, enabling in situ root phenotyping. An indoor experiment was conducted, involving three growth mediums (natural soil, TS, hydroponics), two fibrous-root crop species (wheat, maize) and two taproot crop species (cotton, soybean), to evaluate the impact of TS on root morphology and water and nitrogen uptake of crops with different root types. Results showed that, compared with the average difference between hydroponics and natural soil, the average difference in root morphology and water and nitrogen uptake of maize between TS and natural soil was significantly decreased, as well as for cotton, soybeans, and wheat in turn. It was concluded that compared to those developed in hydroponics, the root developed in TS was significantly more similar to those developed in natural soil. Yet such similarity varied across crop species, with no clear correlation to root types. These findings provide a theoretical foundation for promoting the application of TS.

## 1. Introduction

Variations in root system architecture (RSA) can significantly impact crop productivity under different environments [1,2]. Due to the lack of sensing mechanisms that extend beyond the plant itself, roots form branched three-dimensional (3D) networks, foraging blindly in the soil and adapting their developmental programs in response to environmental stimuli, such as locally encountered resource patches [3]. The timing and location of root growth largely determine a plant’s ability to acquire water and nutrients, which in turn are constrained by genetically encoded developmental programs and environmental perception [4]. Therefore, identification, evaluation, and selection of root traits require innovations that enable high-throughput and accurate quantification of RSA [5,6].

Characterizing the RSA in the laboratory is clearly constrained by a necessary compromise. On one hand, media that are representative of field soil (such as soil and sand) are opaque to most forms of radiation and have limited ability to control the heterogeneous factors that affect root development (e.g., gradients in water availability, nutrient concentrations, and porosity). On the other hand, transparent media (such as hydroponics, aeroponics, and gels) cannot provide field-relevant phenotypes and growing conditions [7]. Soil-based Rhizobox is a common in situ 2D root observation platform in the laboratory with high resolution [8], but only the root systems growing on the transparent surface could be obtained. In recent years, many breakthroughs in the extraction and analysis methods of 2D root under soil-based Rhizobox have been made [9,10]. X-ray computed tomography (CT) and magnetic resonance imaging, often regarded as the state-of-the-art technique, allow for the 3D reconstruction of RSA in the soil of PVC tube [11,12]. Yet there are significant imaging and image processing challenges to the recovery of intact root systems using X-ray CT [13,14,15]. Due to the characteristics of CT system performance, the contradiction between sample size and image resolution is still a key issue. In addition, algorithms for extracting root systems from CT images still have many problems in practical applications.

As mentioned earlier, in addition to soil and sand, transparent media such as hydroponics and gels also allow for acquisition in 2D or 3D of RSA in the laboratory [4,16,17,18]. Such methods have advantages such as high throughput and image resolution. However, due to the significant differences in properties between transparent media and natural soil, the generalization of the obtained results is often a cause for concern. To address this issue, Ma et al. (2019) [7] developed a transparent soil (TS) formed by the spherification of hydrogels of biopolymers, with high transparency, good mechanical stability, tunable pore sizes and easy scalability. This porous media can support root growth in the presence of air, water, and nutrients, and allows for the imaging of unconstrained root systems in vivo by both photography and microscopy [19]. To enable this porous medium wider utilization, Li et al. (2023) [20] further optimize the reagent cost of TS. Of course, this TS medium has limitations. The main limitations are listed as follows: (1) the transparency and the mechanical properties limit the size of the root volume; (2) the surface chemistry of the TS beads is significantly different from that of soil. In summary, when all methodologies have limitations, developing complementary techniques with different limitations is usually the best option. In this sense, this TS fills a much-needed niche in the spectrum of techniques available for root phenotyping. Although TS has promising application prospects in the field of root phenotyping, the evaluation studies of its impact on root morphology and water and nitrogen uptake in crops with taproot and fibrous roots are still scarce.

For this study, the representative fibrous-rooted field crops, including wheat and maize, and tap-rooted field crops, namely, cotton and soybeans, were chosen as subjects. Besides the TS, natural soil and hydroponics were also established, totaling three growth medium treatments. The research objective was to investigate the impact of TS on root morphology and water and nitrogen uptake of wheat, maize, cotton, and soybeans using natural soil and hydroponics as controls and to confirm that the roots developed in TS are significantly more similar to those developed in natural soil than those developed in hydroponic conditions. This study verified the hypotheses that compared with hydroponics; there was no significant difference in the morphological characteristics or water and nitrogen absorption patterns of wheat, corn, cotton, and soybean between natural soil and TS. The results of our study can provide a theoretical basis for the further application of TS, which is a novel plant growth medium.

## 2. Results and Discussion

### 2.1. Plant Growth

Previous studies indicate that the transparent soil has a particle size of 2.6–5.3 mm, and collapse stress values ranging from 0.35 to 3.72 Kpa (Li et al., 2023) [20]. Therefore, transparent soil has a certain level of support capacity for crop root growth. However, the dry weights of crops exhibit distinct responses to transparent soil. As shown in Table 1, except for maize, which showed no significant differences in root dry matter weight among the three substrates, transparent soil significantly reduced the root dry matter weights of wheat, cotton, and soybean. Specifically, compared with natural soil, the root dry matter weights of wheat, cotton, and soybean in transparent soil decreased significantly by 44.8%, 38.5%, and 46.1%, respectively (Table 1). In comparison with the hydroponics where roots receive no support, transparent soil significantly reduced the root dry matter weight of wheat and cotton, with an average reduction of 33.3%. Analysis of variance indicated that the growth substrate had significant effect on taproot root dry weight (Table 2). However, unlike root dry weight, the aboveground dry weight was only significantly affected in wheat grown in transparent soil. Compared with natural soil and hydroponics, the aboveground dry matter weight of wheat in transparent soil decreased by 18.5% and 24.1%, respectively (Table 1). The analysis of the root–shoot ratio indicated that there was no significant difference in maize among the three growth substrates. For wheat, cotton, and soybean, however, transparent soil significantly reduced their root–shoot ratio compared with natural soil, while no significant differences were observed between transparent soil and hydroponics. The root–shoot ratio of the taproot system was significantly affected by the growth substrate but not significantly influenced by crop species (Table 2).

The decreases in root–shoot ratio under transparent soil appears to be mediated by promoted aboveground growth and inhibited root growth compared to natural soil. These results differ slightly from previous studies. Wojciechowski et al. (2009) [21] found that gel chambers promoted increases in root dry weight compared to field conditions but inhibited increases in aboveground dry weight of wheat, thereby increasing the root-to-shoot ratio. However, Ma et al. (2019) [7] found no significant differences in soybean root and aboveground dry weight between transparent soil and soil, and the root-to-shoot ratio was not affected by the growing substrates. Meanwhile, there were significant differences in dry weight between crop species (Table 2). This discrepancy may stem from the fact that crop growth is regulated not only by genetic regulation but also by external environmental conditions. Therefore, variations in experimental conditions and crop varieties can lead to differences in the results obtained.

### 2.2. Root Morphology

#### 2.2.1. Global Traits

**Figure 1:**
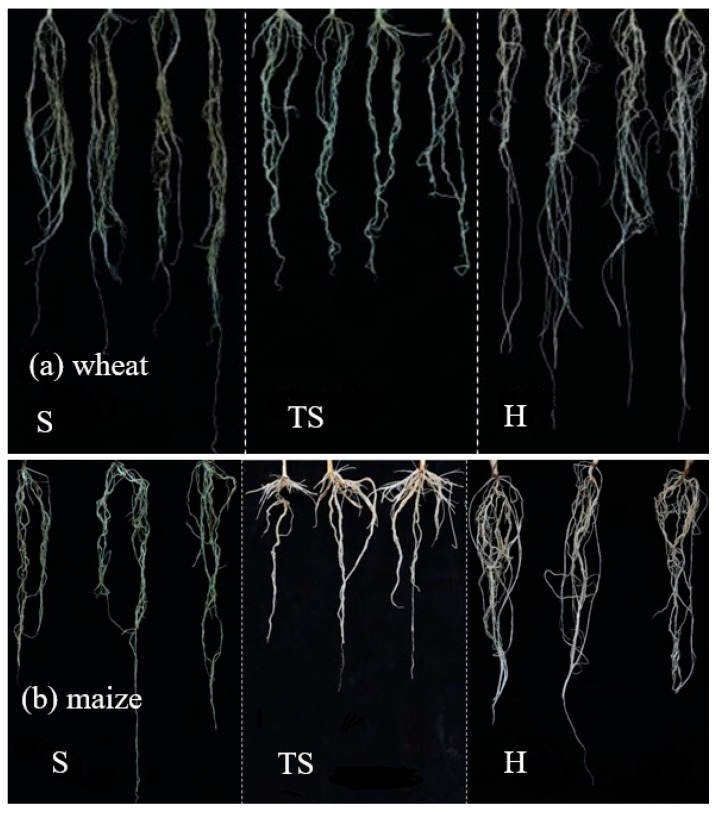
Wheat (**a**) and maize (**b**) root phenotyping after 9 and 10 days grown in different growth substrates. S, natural soil; TS, transparent soil; H, hydroponic culture.

**Figure 2:**
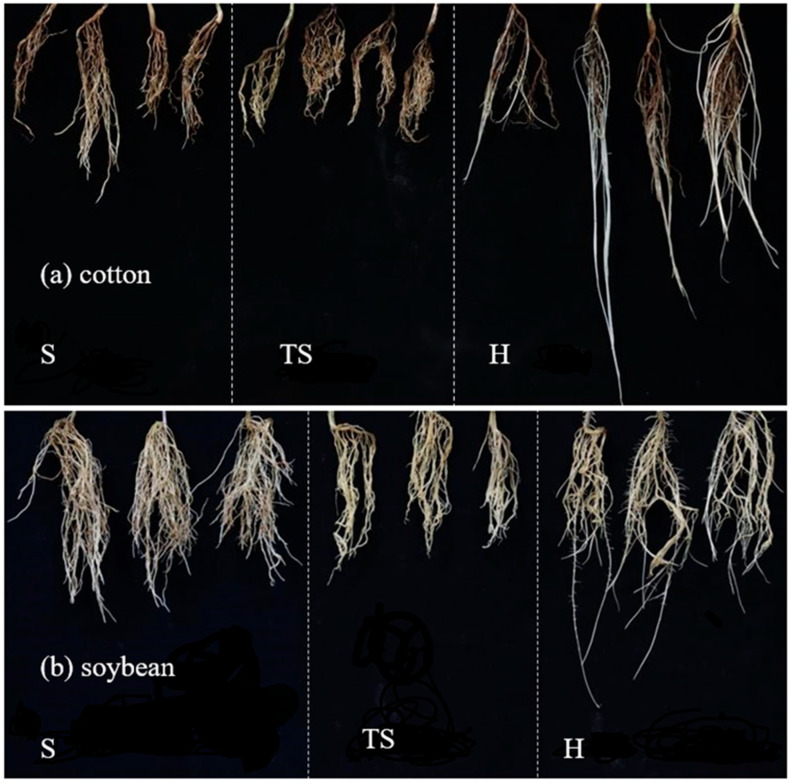
Cotton (**a**) and soybean (**b**) root phenotyping after 9 and 10 days grown in different growth substrates. S, natural soil; TS, transparent soil; H, hydroponic culture.

The root architecture developed in transparent soil was visibly more similar to that developed in natural soil than in hydroponic conditions (Figure 1 and Figure 2). Meanwhile, a comparison of root morphological characteristics in different growth substrates shows that the root architecture in transparent soil is closer to that developed in natural soil than in hydroponic conditions, especially maize.

**Figure 3:**
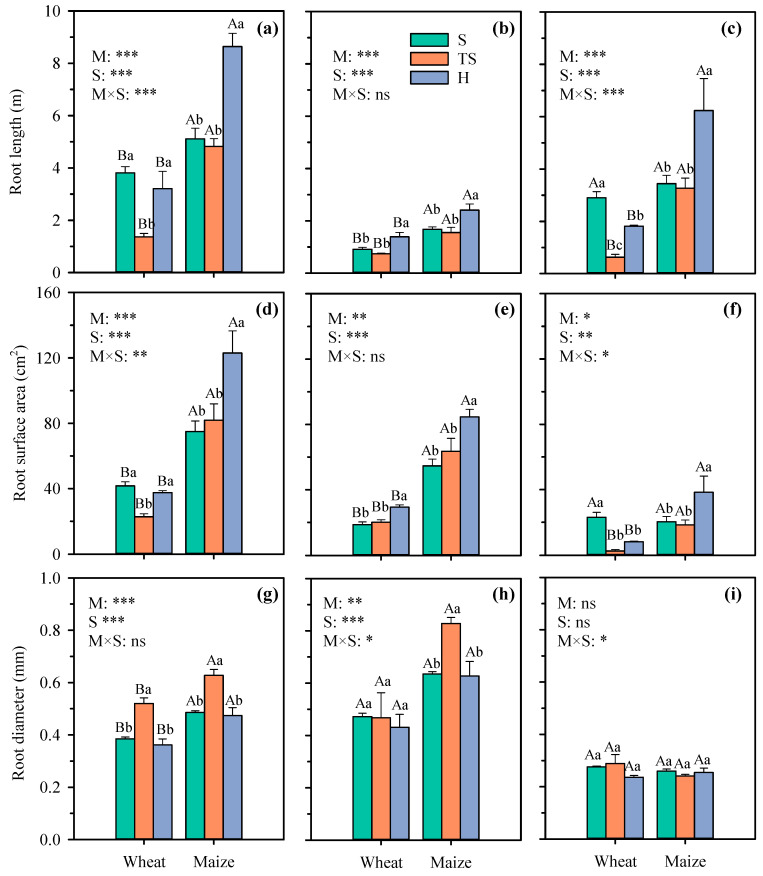
The root length, root surface area, and root diameter of total roots (**a**,**d**,**g**), adventitious (**b**,**e**,**h**), and lateral (**c**,**f**,**i**) roots of wheat and maize under different growth substrates. Each value represents the mean ± standard error of 3 replicates. Bars labeled with the same capital letters show no significant difference (*p* < 0.05) between species for each growth substrate. Bars labeled with the same lowercase letters show no significant difference (*p* < 0.05) between three growth substates for each crop. * Significant at 0.05 probability level; ** significant at 0.01 probability level; *** significant at 0.001 probability level; ns: not significant. S, natural soil; TS, transparent soil; H, hydroponic culture. M, growth medium; S, crop species.

**Figure 4:**
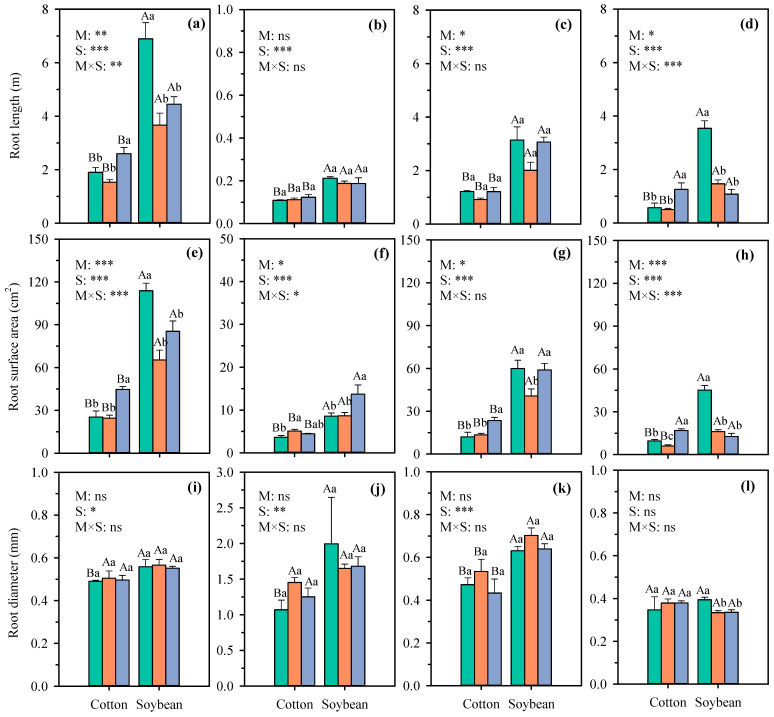
The root length, root surface area, and root diameter of total (**a**,**e**,**i**), taproots (**b**,**f**,**j**), first-order lateral (**c**,**g**,**k**), and second-order lateral (**d**,**h**,**l**) roots in cotton and soybean under different growth substrates. Each value represents the mean ± standard error of 3 replicates. Bars labeled with the same capital letters show no significant difference (*p* < 0.05) between species for each growth substrate. Bars labeled with the same lowercase letters show no significant difference (*p* < 0.05) between three growth substates for each crop. * Significant at 0.05 probability level; ** significant at 0.01 probability level; *** significant at 0.001 probability level; ns: not significant. S, natural soil; TS, transparent soil; H, hydroponic culture. M, growth medium; S, crop species.

The root length, root surface area, and root diameter of different crop grown in three growth substrates are presented in Figure 3 and Figure 4. As can be seen from the results, both fibrous root and taproot systems exhibit distinct responses to different growth substrates, with variations also observed among crops of the same type. For example, compared with natural soil, transparent soil reduced the root length of wheat, maize, cotton, and soybean by 64.3%, 5.7%, 19.5%, and 46.8%, while the root surface of wheat, maize, cotton, and soybean reduced by 45.5%, −9.3%, 2.7%, and 42.6%, respectively. However, significant decreases were detected in root length and root surface area of wheat and soybean. Further analysis revealed that the reductions in root length and root surface area of soybean and wheat in transparent soil (vs. natural soil) were primarily attributed to the decreased development of the first and secondary lateral in soybean and the adventitious roots in wheat (Figure 3c,f and Figure 4d,h). Compared with natural soil, transparent soil reduced the lateral root length and surface area of soybean average by 47.2% and 48.4%, while the length and surface area of lateral roots of wheat decreased by 78.4% and 88.9%, respectively. Whereas there were no significant differences in root length of maize and cotton between natural soil and transparent soil. Nevertheless, growth substrates and crop species had significant effects on root length and root surface area.

This result is consistent with Downie et al.’s (2012) [22] research on maize and alfalfa and tobacco. Owing to the favorable root growth environment in hydroponics, root length and root surface area increased in all crops compared to transparent soil. Compared with hydroponics, transparent soil decreased the root length of wheat, maize, cotton, and soybean by 57.5%, 44.2%, 17.5%, and 41.0%, while the root surface of wheat, maize, cotton, and soybean reduced by 39.4%, 33.4%, 23.5%, and 45.0%, respectively. Further analysis revealed that the reduction in root length and root surface area in transparent soil compared with hydroponics was primarily due to the significant reduction in the secondary lateral roots of cotton and the lateral adventitious roots of maize and wheat (Figure 3c,f and Figure 4d,h). There were no significant differences in root length of soybean between transparent soil and hydroponics. Root diameter of wheat and maize in transparent soil was significantly higher than that of hydroponics and natural soil, especially the adventitious root diameter of maize, which increased its average by 32%. Although growth substrates had significant effect on root length and root surface area, it is noteworthy that compared with hydroponics, the root parameters of maize and cotton in transparent soil exhibited less variation than those in natural soil.

Root architecture determines the ability of crops to acquire resources such as water and nutrients and is influenced by both genetic and environmental factors [23]. The results of this study differ from those of Ma et al. (2019) [7] on soybean, which may be attributed to differences in the varieties used and growth environmental conditions. Regarding the effects of substrates on root growth, Wojciechowski et al. (2009) [21] found that gel chamber wheat roots were longer than in soil, attributed to differences in nutrient availability in the experiment. Robinson (1994) [24] noted that low nutrient conditions in gel may promote increased root length. However, both hydroponic culture and transparent soil in this study used half-strength modified Hoagland nutrient solution, which has higher nutrient availability than natural soil. Therefore, nutrient availability is not the reason for root growth. Hargreaves et al. (2009) [25] found that gel increases barley root length due to higher mechanical resistance in soil. The dry bulk density of natural soil in this study was 1.45 g cm^−3^, which was higher than the 1.1 g cm^−3^ observed in the experiment conducted by Hargreaves et al.’s (2009) [25] study. However, the results show that the root length of wheat and soybean growth in natural soil was longer than in transparent soil, suggesting that mechanical resistance may not be the factor causing root changes. Clark et al. (1999) [26] found that the reduction in root length in gel was not due to mechanical inhibition and these inhibitory effects were not observed when using a new batch of experimental materials. Most root parameters were affected by growth substrate and crop species. Therefore, the differences in root length and root surface area among different crops in different treatments in this study may be the result of crop adaptation to the growth substrates.

**Figure 5:**
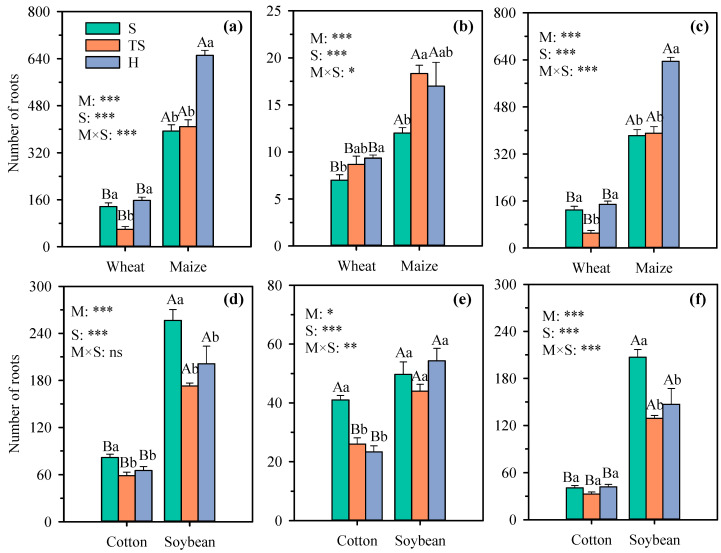
Number of total roots (**a**), first-order lateral (**b**), and second-order lateral roots (**c**) in cotton and soybean, along with adventitious (**d**), latera (**e**), and total roots (**f**) in wheat and maize under different growth substrates. Each value represents the mean ± standard error of 3 replicates. Bars labeled with the same capital letters show no significant difference (*p* < 0.05) between species for each growth substrate. Bars labeled with the same lowercase letters show no significant difference (*p* < 0.05) between three growth substates for each crop. * Significant at 0.05 probability level; ** significant at 0.01 probability level; *** significant at 0.001 probability level; ns: not significant. S, natural soil; TS, transparent soil; H, hydroponic culture. M, growth medium; S, crop species.

Research shows that the abundant lateral roots of crop root can increase their absorption area in the soil, thereby effectively obtaining soil resources [27]. Compared to natural soil, only the total root number of maize grown in transparent soil remained unchanged, while the root number of cotton, soybean, and wheat decreased significantly by 28.2%, 32.6%, and 56.4%, respectively (Figure 5a,d). Further analysis indicated that the reduction in soybean root numbers was due to a significant decrease of 37.7% in secondary lateral roots, cotton was due to a significant reduction of 36.6% in primary lateral roots, and wheat was due to a significant decrease of 60.8% in lateral roots. Meanwhile, compared with hydroponics, transparent soil had no significant difference in root numbers for taproot systems but significantly reduced the total root number of fibrous root systems, especially with a significant decrease in lateral root numbers (Figure 5c). Compared with hydroponics, the number of lateral roots of wheat and maize decreased by 65.6% and 38.5%, respectively.

Downie et al. (2012) [22] studied the effects of transparent polymer Nafion as a growth substrate on lettuce root systems, finding that root growth in transparent substrates with pore structures and mechanical support was similar to root growth in soil. The results of this study indicate that compared to hydroponics, the root length, surface area, and root diameter growth in transparent soil are more similar to those in soil, especially for maize and cotton.

#### 2.2.2. Local Traits

SRL is an important indicator reflecting the ability of roots to absorb water and nutrients [28]. The effects of different growth substrates on crop SRL vary significantly. Compared with natural soil, the SRL of wheat and maize in transparent soil decreased by 32.5% and 16.1%, whereas in cotton and soybean, it increased by 29.9% and 0.42%, respectively. Simultaneously, compared with hydroponics, transparent soil reduced the SRL of wheat, maize, cotton, and soybean by 32.9%, 28.2%, 34.53%, and 1.7%, respectively. There were no significant differences in the SRL of maize and soybean among different growth substrates, indicating that there was no difference in root uptake ability among the three growth substrates. Wheat and soybean have a closer SRL to the natural soil.

Root tissue density (RSD) is the mass of roots per unit volume and reflects the ability of roots to acquire resources and defend against stresses [29]. Previous studies have shown that RSD decreases with increasing nutrient availability [30]. In this study, the RSD of four crops grown in transparent soil was lower than in natural soil (only maize did not reach significant level), and there was no significant difference from hydroponics (Table 3). Compared with natural soil, transparent soil reduced the RSD of wheat, maize, cotton, and soybean by 29.8%, 23.3%, 29.2%, and 24.4%, respectively. The same culture solution was used in hydroponics and transparent soil, so there was no significant difference in RSD; that is, the RSD of taproot in TS is similar to that in hydroponics. RSD is significantly affected by the growth substrate.

The growth and development of lateral roots are affected by growth substrates (Table 3). Compared with natural soil, transparent soil reduced the lateral root density of wheat and soybean by 51.4% and 32.2%, whereas in maize and cotton, it increased by 12.2% and 6.9%, respectively. For wheat, both the length and number of lateral roots in the transparent soil were significantly lower than in natural soil, resulting in a reduction in lateral root density. For soybean, the length and number of secondary lateral roots in transparent soil were significantly lower than in natural soil, leading to a reduction in lateral root density. Compared with hydroponics, transparent soil reduced the lateral root density of wheat, maize, cotton, and soybean by 30%, 4.2%, 9.0%, and 31.1%, respectively. This is consistent with Downie et al.’s (2012) [22] research findings that the lateral root density in transparent soil is lower than that in natural soil.

Due to differences in root architecture, there were no significant differences in the distribution of lateral roots among growth substrates for fibrous root systems, but significant variations were observed in that of taproot systems. Compared with natural soil, transparent soil reduced the distribution of lateral roots of wheat, maize, cotton, and soybean by 46%, 22.9%, 42%, and 45.7%, respectively. Compared with hydroponics, transparent soil reduced the distribution of lateral roots of wheat, maize, cotton, and soybean by 35.4%, 6%, 47%, and 52%, respectively. The results of Downie et al. (2012) [22] indicate that the density of lateral roots of transparent soil plants is lower than in soil and sand cultures. Other studies suggest that the distinct differences of lateral roots between gel and soil may be attributed to interactions between root hairs and gel or root responses to chemical substances in transparent soil [26]. Further research is required to explore the mechanisms underlying the effect of transparent soil on lateral root growth.

### 2.3. Water and N Uptake

**Figure 6:**
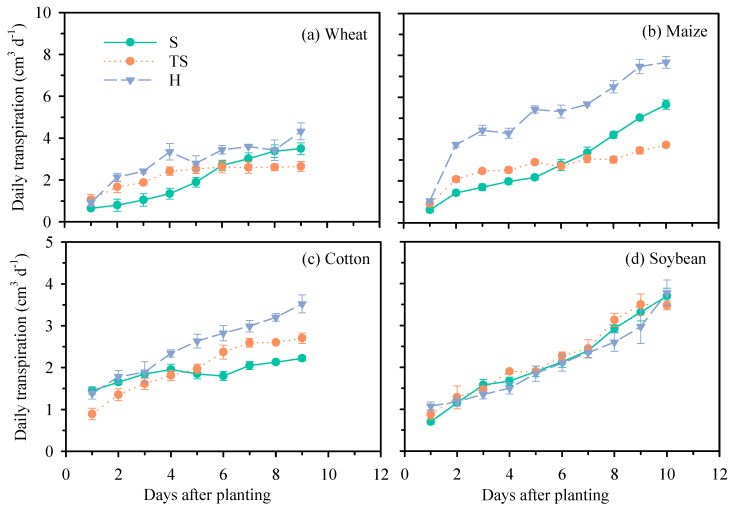
Daily transpiration of different crops under different growth substrates during the experiment period. (**a**): wheat; (**b**): maize; (**c**): cotton; (**d**): soybean. Each value represents the mean ± standard error of 3 replicates. S, natural soil; TS, transparent soil; H, hydroponic culture.

As the main organ of plants for acquiring water and nutrients, the growth and development of roots directly affects the uptake of water and nutrients. The daily transpiration dynamics of four crops under different growth substrates are shown in Figure 6. It can be seen from the figure that, except for soybean, the daily transpiration of cotton, maize, and wheat under hydroponic culture is higher than natural soil and transparent soil, which is consistent with previous research results [31,32]. Compared with natural soil, the daily transpiration of maize, wheat, and cotton growth in transparent soil increased by an average of 9.6%, 33.6%, and 3.6%, respectively (Figure 6). Compared with hydroponics, the daily transpiration of maize, wheat, and cotton growth in transparent soil decreased by an average of 44.2%, 20%, and 21.4%, respectively (Figure 6). Relative to hydroponics, the water transpirations of crops grown in transparent soil are closer to those under natural soil.

**Figure 7:**
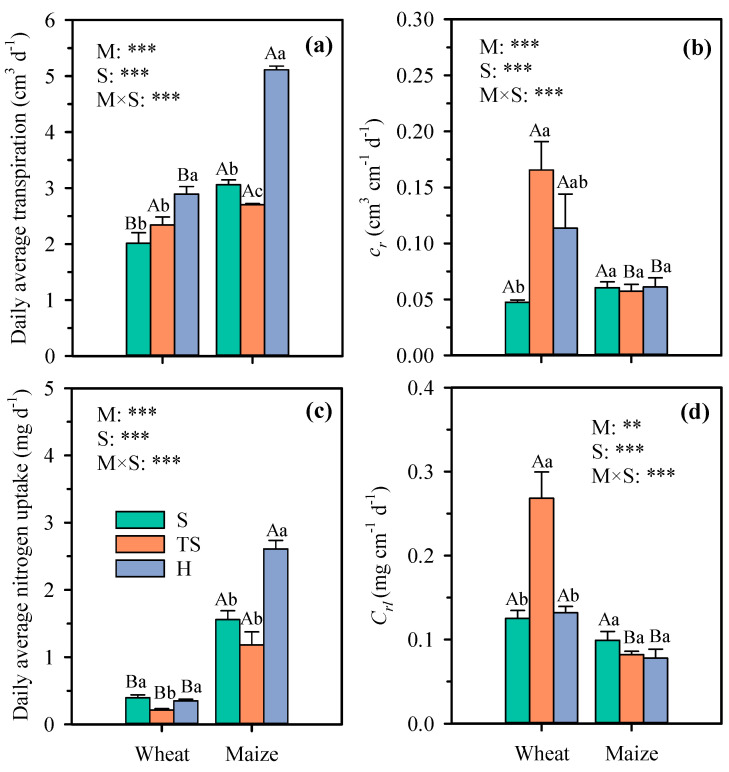
Daily average transpiration (**a**), root water uptake per unit length (**b**), daily average nitrogen uptake (**c**), and root nitrogen uptake per unit length (**d**) of wheat and maize under different growth substrates. Each value represents the mean ± standard error of 3 replicates. Bars labeled with the same capital letters show no significant difference (*p* < 0.05) between species for each growth substrate. Bars labeled with the same lowercase letters show no significant difference (*p* < 0.05) between three growth substates for each crop. ** significant at 0.01 probability level; *** significant at 0.001 probability level. S, natural soil; TS, transparent soil; H, hydroponic culture. M, growth medium; S, crop species.

However, analysis of daily average transpiration revealed that compared with natural soil, wheat grown in transparent soil exhibited a 16% increase, whereas that of maize decreased by 12%. Although maize daily average transpiration and root length significantly increased under hydroponic culture, the potential water uptake coefficient per unit root length showed no significant differences with natural soil and transparent soil (Figure 7b). At the same time, there was no significant difference in *c_r_* between hydroponic culture and natural soil, which is consistent with previous studies on wheat and rice [33,34]. The shorter root length of wheat resulted in root water uptake capacity that was 232% higher than in natural soil and 48.1% higher than in hydroponics. The analysis of daily nitrogen uptake indicated that different growing substrates influenced crop nitrogen uptake characteristics. Results showed that maize exhibits no significant differences in daily nitrogen uptake and root nitrogen uptake capacity between transparent soil and natural soil (Figure 7c,d).

**Figure 8:**
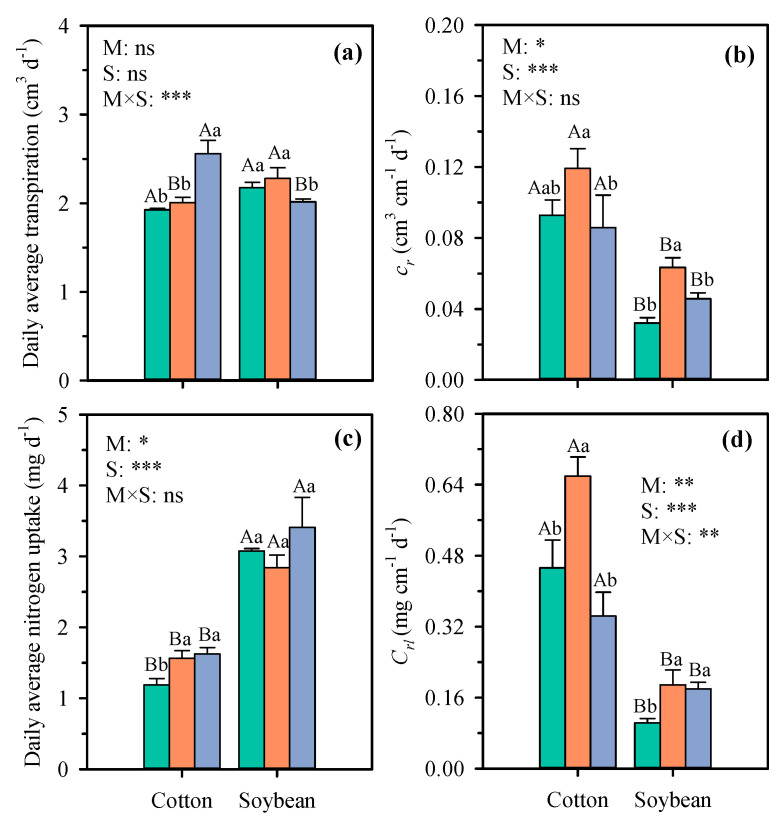
Daily average transpiration (**a**), root water uptake per unit length (**b**), daily average nitrogen uptake (**c**), and root nitrogen uptake per unit length (**d**) of cotton and maize under different growth substrates. Each value represents the mean ± standard error of 3 replicates. Bars labeled with the same capital letters show no significant difference (*p* < 0.05) between species for each growth substrate. Bars labeled with the same lowercase letters show no significant difference (*p* < 0.05) between three growth substates for each crop. * Significant at 0.05 probability level; ** significant at 0.01 probability level; *** significant at 0.001 probability level; ns: not significant. S, natural soil; TS, transparent soil; H, hydroponic culture. M, growth medium; S, crop species.

Although there was no significant difference in the daily average transpiration of cotton and soybean between natural soil and transparent soil, the root water uptake capacity in transparent soil is higher than in natural soil. Compared with natural soil, *cr* in transparent soil of cotton and soybean increased by 22.1% and 49.4% (Figure 8b). Nevertheless, transparent soil grows cotton and soybean with higher *c_r_* than hydroponic culture and natural soil (Figure 8b). Compared with hydroponics, *cr* in transparent soil of cotton and soybean average decreased by 38.7% (Figure 8b). For cotton and soybean, both hydroponics and transparent soil can provide adequate nitrogen nutrition, so there is no significant difference in daily nitrogen uptake (Figure 8c), but only soybean shows no significant difference in *C_rl_* compared to hydroponic conditions (Figure 8d). In summary, root absorption performance is significant influenced by the cultivation substrate, and only maize shows no significant difference in root absorption performance across the three cultivation substrates.

**Figure 9:**
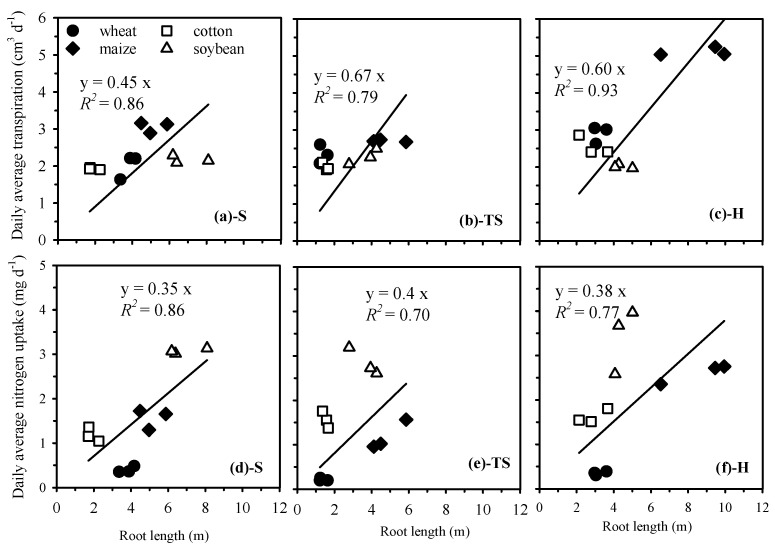
The relationship between daily average transpiration and root length of natural soil (**a**), transparent soil (**b**), and hydroponics (**c**) and the relationship between daily average nitrogen uptake and root length of natural soil (**d**), transparent soil (**e**), and hydroponics (**f**).

As shown in Figure 9, both the transpiration and nitrogen uptake are significantly positively correlated with root length, which is consistent with the findings of previous studies [34]. Although transparent soil can provide a certain degree of mechanical support for root systems, the ratio of root length to nitrogen and water uptake is closer to that under hydroponics (Figure 9).

## 3. Materials and Methods

### 3.1. Experiment Design

The experiment was conducted in the laboratory at the Qiliying Comprehensive Experimental Station of the Chinese Academy of Agricultural Sciences (35°54′ N, 113°29′ E) from November 2023 to June 2024. Wheat (*Triticum aestivum* L. cv. kelin 618), maize (*Zea mays* L. cv. Yufeng 303), cotton (*Gossypium hirsutum* L. cv. xinluzhong 37), and soybean (*Glycine max* L. cv. zhonghuang 301) were used as plant materials. The three growth substrate treatments were natural soil, transparent soil, and hydroponics, each with three replicates.

The natural soil was collected from the 0~20 cm plough layer of the Qiliying Comprehensive Experimental Station of the Chinese Academy of Agricultural Sciences. The soil had 45.9 mg kg^−1^ available nitrogen, 18.5 mg kg^−1^ phosphorus, and 134.8 mg kg^−1^ potassium contents. The soil was first air-dried and sieved, then filled in layers at 5 cm intervals with a bulk density of 1.45 g cm^−3^, resulting in a total soil depth of 15 cm after filling. Then, 1.32 g water-soluble compound fertilizer (15% pure N, 5% P_2_O_5_, and 30% K_2_O) was applied at a rate equivalent to 750 kg ha^−1^ to ensure adequate nutrition for crops. Finally, the soil was irrigated to 80% of field capacity for sowing preparation. The particle size distribution, texture, and hydraulic properties of soil are shown in Table 4.

The brief procedure for making TS is as follows: (1) Prepare the polymer solution with a concentration of 1.2 g L^−1^ (the phytagel and sodium alginate power were mixed with a ratio of 4:1, wt). (2) Prepare MgCl_2_ solution with a concentration of 10 mmol L^−1^ as the cross linker solution. (3) Prepare the polymer solution and MgCl_2_ solution in a volume ratio of 1:5. (4) Using a self-made simple device, drop the polymer solution into the solution of MgCl_2_ rapidly and gel the polymer into discrete spherical beads. The detailed process of TS particles refers to Li et al. (2023) [33]. To align the porosity of TS with those of natural soil as closely as possible, the inner diameter of the nozzle was set to 1.5 mm according to the findings of Ma et al. [7]. The diameter of TS particles was approximately 4.9 mm, and the measured total porosity was about 0.334, which is very close to the field capacity moisture content of 0.340 cm^3^ cm^−3^ for natural soil (Table 4). The prepared TS particles were soaked in the modified Hoagland nutrient solution (NSP1020, Coolaber, Beijing, China) for more than 3 h at a volume ratio of 1:1 (transparent soil: nutrient solution) until equilibration, to make TS particles containing half-strength nutrient solution [7]. The TS was then evenly distributed into PVC tubes. To ensure that the nutritional conditions are as consistent as possible with TS treatment, the same half-strength modified Hoagland nutrient solution was used for hydroponic treatment.

All seeds were subjected to careful disinfection to minimize their mold growth during the TS treatment, with the specific steps outlined as follows: (1) cleaned with deionized water, then soaked in 30% hydrogen peroxide for 20 min, and rinsed 4 times with deionized water; (2) soaked in 2.5% sodium hypochlorite solution for 15 min, followed by rinsing with deionized water with three times. After the disinfection was completed, all the seeds were transferred to sterile paper saturated with deionized water and placed in an incubator at 30 °C until germination (radicle emergence). For both natural soil and transparent soil treatments, plump and uniform seeds were selected. Each PVC bucket (20 cm in diameter, 20 cm in height) was sown with 3 seeds evenly. After growing to the 2-leaf stage, thinning was performed to retain one seedling per bucket. For the hydroponic treatment, germinated seeds were first transferred to quartz sand irrigated with half-strength modified Hoagland nutrient solution. After reaching the 2-leaf stage, seedlings of uniform size were selected; their roots were rinsed with deionized water and then transferred to nutrient solution with sponge fixation. During the crop growth period, continuous light was provided daily for 12 h (from 06:00 to 18:00), the effective light intensity at the plant canopy was 400 μmol m^−2^ s^−1^, and the day/night temperatures were 25/20 °C, respectively.

For the natural soil treatment, irrigation was performed with the amount of evapotranspiration loss to maintain the soil water content at 80% of field capacity daily. For the TS treatment, full-strength modified Hoagland’s nutrient solution was applied every 3 day and then drained after soaking for 3–4 h to ensure the nutrient level of TS was maintained at half-strength. For the hydroponic treatment, the nutrient solution was replaced every 3 days, and fresh air was injected continuously with a compressor to alleviate oxygen insufficiency. The comparison of physical and chemical properties and root growth condition of natural soil, TS, and hydroponics is shown in Table 4. The half-strength modified Hoagland solution (pH = 7.8) contained (mg L^−1^) KNO_3_—253, NH_4_NO_3_—40, KH_2_PO_4_—78, MgSO_4_—120.5, FeNaEDTA—18.35, KI—0.415, H_3_BO_3_—3.1, MnSO_4_·H_2_O—11.3, ZnSO_4_·7H_2_O—4.3, Na_2_MoO_4_·2H_2_O—0.125, CuSO_4_·5H_2_O—0.0125, CoCl_2_·6H_2_O—0.0125, Ca(NO_3_)_2_·4H_2_O—472.5.

### 3.2. Measurements and Methods

Daily transpiration (cm^3^ d^−1^) was determined by measuring weight changes at 8:30 am each day. Since each PVC bucket was covered with a PVC lid, evaporation was assumed to be absent during the experiment. At the end of the experiment, leaf and root were scanned using a scanner (Epson Perfection V800 Photo, Epson (China) Co., Ltd. Beijing, China), and the leaf images were analyzed with ImageJ software (ImageJ 1.54, NIH, Bethesda, MD, USA) to determine leaf area (cm). The root images were analyzed by RhizoVision Explorer 2.0.3 [35] to determine parameters including root length, root surface area, number of branched roots, position of branched roots, branching density, and average diameter. The dry weights of root and aboveground organs were determined by drying for 48 h to a constant at 75 °C. The dried samples were then ground and nitrogen content was measured with an elemental analyzer (CHNSO EA 1108, Carlo Erba reagents, Milan, Italy). The calculation formulas for other indicators are as follows:

(1) Root to shoot ratio (RSR, g g^−1^) = root dry weight (g)/shoot dry weight (g)

(2) Specific Root Length (SRL, m/g) = root length (m)/root dry weight (g)

(3) Root tissue density (RSD, g cm^−3^) = root dry weight (g)/root volume (cm^3^)

(4) Lateral root density (LRD, No. cm^−1^) = lateral root number/parental root length

(5) Lateral root distribution ratio (LRDR, %) = lateral root distribution range (cm)/parental root length (cm)

(6) The potential root water uptake coefficient (*c_r_*, cm^3^ cm^−1^ d^−1^) = the daily average transpiration (cm^3^ d^−1^)/root length (cm)

(7) The potential root nitrogen uptake coefficient (*C_rl_*, mg cm^−1^ d^−1^) = the daily average nitrogen uptake (mg d^−1^)/root length (cm)

### 3.3. Statistical Analysis

Analysis of variance (ANOVA) was performed with the general linear model (GLM) procedure in IBM SPSS Statistics for windows, version 20.0 (IBM Corp., Armonk, NY, USA). One-way ANOVA was performed to evaluate the effects of a variable on the studied parameters based on least significant differences (LSDs) at 0.05 probability level. A multifactor ANOVA was conducted to explore the interaction of various variables, such as growth substrates and crop species. Graphs were produced using Sigmaplot 14.0 (Systat Software, San Jose, CA, USA).

## 4. Conclusions

To further promote the application of transparent soil in crop root phenotyping research, this study systematically compared differences in root morphology and water–nitrogen uptake performance among four crops (wheat, maize, cotton, and soybean) grown in natural soil, transparent soil, and hydroponic conditions. The results showed that, compared with hydroponic conditions, crop root morphology and function under transparent soil conditions were more similar to those in natural soil. However, this similarity varied among crops: maize exhibited the highest similarity overall, followed by cotton and soybean, while wheat showed the lowest similarity. Although transparent soil improves the representativeness of crop root phenotyping results compared to hydroponics, further in-depth research is still needed to explore the influence of transparent soil on the three-dimensional root system architecture of crops and the underlying physiological mechanisms.

## Figures and Tables

**Table 1:** Root and shoot dry weight and root–shoot ratio of maize, wheat, cotton, and soybean under different growth substrates.

Parameters	Treatments	Fibrous Root System		Taproot System	
		Maize	Wheat	Cotton	Soybean
Root dry weight (g)	S	0.056 ± 0.001 ^Aa^	0.029 ± 0.003 ^Ba^	0.026 ± 0.003 ^Ba^	0.089 ± 0.003 ^Aa^
	TS	0.062 ± 0.011 ^Aa^	0.016 ± 0.002 ^Bb^	0.016 ± 0.001 ^Bb^	0.048 ± 0.003 ^Ab^
	H	0.064 ± 0.005 ^Aa^	0.024 ± 0.001 ^Ba^	0.024 ± 0.004 ^Ba^	0.057 ± 0.004 ^Ab^
Aboveground dry weight (g)	S	0.254 ± 0.004 ^Ab^	0.054 ± 0.004 ^Ba^	0.116 ± 0.005 ^Bb^	0.355 ± 0.011 ^Aa^
	TS	0.233 ± 0.016 ^Ab^	0.044 ± 0.001 ^Bb^	0.141 ± 0.014 ^Bab^	0.381 ± 0.011 ^Aa^
	H	0.321 ± 0.014 ^Aa^	0.058 ± 0.002 ^Ba^	0.149 ± 0.006 ^Ba^	0.368 ± 0.017 ^Aa^
RSR	S	0.219 ± 0.004 ^Ba^	0.540 ± 0.03 ^Aa^	0.225 ± 0.04 ^Ba^	0.25 ± 0.001 ^Aa^
	TS	0.275 ± 0.038 ^Aa^	0.369 ± 0.029 ^Ab^	0.115 ± 0.018 ^Bb^	0.126 ± 0.007 ^Ab^
	H	0.213 ± 0.035 ^Ba^	0.419 ± 0.047 ^Aab^	0.162 ± 0.019 ^Bab^	0.155 ± 0.014 ^Ab^

Note: RSR: root to shoot ratio. Values are shown as the mean ± standard error of three replicates. Different capital letters denote significant difference (*p* < 0.05) between crop species for each growth substrate; different lowercase letters denote significant difference (*p* < 0.05) between three growth substrates for each crop. S, natural soil; TS, transparent soil; H, hydroponic culture.

**Table 2:** *F*-statistics to assess the effect of growth medium (M) and crop species (S) on root growth and root morphology.

Parameters	Fibrous Root System			Taproot System		
	M	S	M × S	M	S	M × S
Root dry weight (g)	0.6 ^ns^	84 ***	2.5 **	34 ***	278 ***	16 **
Aboveground dry weight (g)	16.7 ***	849 ***	9.5 **	3.1 ^ns^	634 ***	0.5 ^ns^
RSR	2.4 ^ns^	64 ***	6.4 *	17 ***	0.3 ^ns^	0.3 ^ns^
SRL (m/g)	12.6 ^ns^	0.7 ^ns^	0.5 ^ns^	3.7 ^ns^	8 *	3.2 ^ns^
Root tissue density (g/cm^3^)	6 *	0.315 ^ns^	0.47 ^ns^	10 **	4.3 ^ns^	0.3 ^ns^
Lateral root density (number cm^−1^ primary root)	5.4 *	220 ***	11 **	1.49 ^ns^	16.3 **	2 ^ns^
Distribution of lateral roots (%)	38 ***	42 ***	4.6 *	49 ***	1.8 ^ns^	0.1 ^ns^

Note: ns, not significant; * significant at 0.05 probability level; ** significant at 0.01 probability level; *** significant at 0.001 probability level. M, growth medium; S, crop species. Same as below.

**Table 3:** Root system parameters of different crops under three growth substrates.

Parameters	Treatments	Fibrous Root System		Taproot System	
		Wheat	Maize	Cotton	Soybean
SRL(m/g)	S	131.0 ± 7.2 ^Aa^	111.0 ± 21.4 ^Aa^	75.22 ± 5.72 ^Ab^	77.5 ± 4.0 ^Aa^
	TS	88.4 ± 13.3 ^Ab^	93.2 ± 21.5 ^Aa^	97.70 ± 0.44 ^Ab^	77.8 ± 13.2 ^Aa^
	H	131.7 ± 7.3 ^Aab^	129.8 ± 23.0 ^Aa^	149.24 ± 23.23 ^Aa^	79.2 ± 7.5 ^Ba^
Root tissue density (g/cm^3^)	S	0.057 ± 0.004 ^Aa^	0.043 ± 0.003 ^Ba^	0.048 ± 0.004 ^Aa^	0.041 ± 0.003 ^Aa^
	TS	0.040 ± 0.004 ^Ab^	0.033 ± 0.002 ^Aab^	0.034 ± 0.004 ^Ab^	0.031 ± 0.002 ^Ab^
	H	0.054 ± 0.005 ^Aab^	0.030 ± 0.005 ^Bb^	0.034 ± 0.005 ^Ab^	0.027 ± 0.003 ^Ab^
Lateral root density (number cm^−1^ primary root)	S	1.43 ± 0.10 ^Ba^	2.29 ± 0.03 ^Aa^	33.32 ± 1.61 ^Ba^	59.98 ± 5.70 ^Aa^
	TS	0.69 ± 0.10 ^Bb^	2.56 ± 0.22 ^Aa^	35.63 ± 0.92 ^Ba^	40.63 ± 5.03 ^Ab^
	H	0.99 ± 0.08 ^Bb^	2.678 ± 0.22 ^Aa^	39.14 ± 3.92 ^Ba^	58.98 ± 4.55 ^Aa^
Distribution of lateral roots (%)	S	64.53 ± 0.68 ^Ba^	75.48 ± 1.38 ^Aa^	49.02 ± 2.78 ^Aa^	45.27 ± 3.61 ^Aa^
	TS	34.84 ± 1.75 ^Bc^	58.18 ± 4.42 ^Ab^	28.42 ± 0.80 ^Ab^	24.57 ± 4.96 ^Ab^
	H	53.9 ± 6.972 ^Bb^	61.89 ± 1.59 ^Ab^	53.62 ± 3.04 ^Aa^	51.35 ± 2.79 ^Aa^

Note: Values are shown as the mean ± standard error of 3 replicates. Different capital letters denote significant difference (*p* < 0.05) between crop species for each growth substrate; different lowercase letters denote significant difference (*p* < 0.05) between three growth substrates for each crop. S, natural soil; TS, transparent soil; H, hydroponic culture.

**Table 4:** The physical and chemical properties and root growth condition of natural soil, TS, and hydroponics.

Growth Medium	Physical and Chemical Properties								Root growth Condition		
	Sand (%)	Silt (%)	Clay (%)	SWC (%)	FC (%)	TP (%)	EP (%)	pH	Water (%)	Nutrition	Oxygen
Natural soil	53.9	42.8	3.3	0.487	0.340			7.8	0.272, Sufficient	Base fertilizer (sufficient)	Sufficient
Transparent soil	Bead diameter: ~4.9 mm			/	/	0.334	0.279	7.8	Sufficient	Half-strength Hoagland (sufficient)	Sufficient
Hydroponics	/			/	/	/	/	7.8	Sufficient	Half-strength Hoagland (sufficient)	Fresh air was injected

Note: SWC: saturated water content; FC: field capacity; TP: total porosity; EP: effective porosity; SWC and FC are related to the volumetric water content.

## Data Availability

The data presented in this study are available on request from the corresponding author. The data are not publicly available due to ongoing research using a part of the data.
